# Effects of Aerobic Dance Exercise and Honey Supplementation Followed by Their Subsequent Cessation on Bone Metabolism Markers and Antioxidant Status in Young Collegiate Females

**DOI:** 10.21315/mjms2023.30.3.14

**Published:** 2023-06-27

**Authors:** Somayeh Sadat Tavafzadeh, Chee Keong Chen, Foong Kiew Ooi, Nurul Azuar Hamzah, Siti Amrah Sulaiman, Jamaayah Meor Osman

**Affiliations:** 1 Exercise and Sports Science Programme, School of Health Sciences, Universiti Sains Malaysia, Kelantan, Malaysia; 2 Pharmacology Department, School of Medical Sciences, Universiti Sains Malaysia, Kelantan, Malaysia

**Keywords:** honey supplementation, aerobic dance, cessation, bone metabolism, antioxidant status

## Abstract

**Background:**

Regular physical activity and proper nutritional intake are crucial for bone health. However, it is unclear if this health benefit is maintained after the removal of these stimuli. This study investigated the combined effects of aerobic dance exercise and honey supplementation, followed by their subsequent cessation on bone metabolism markers and antioxidant status in females.

**Methods:**

Forty-eight young female college students were assigned into four groups: i) 16S (16 weeks of sedentary activity); ii) 8E×8S (8 weeks of exercise followed by 8 weeks of sedentary activity); iii) 8H8S (8 weeks of honey supplementation followed by 8 weeks of sedentary activity) and iv) 8E×H8S (8 weeks of combined exercise and honey supplementation followed by 8 weeks of sedentary activity). Blood samples were collected from the participants prior to the intervention, at week 8 and at week 16 for the analysis of bone metabolism markers and antioxidant status.

**Results:**

At the mid test, bone speed of sound (SOS) (*P* < 0.01), serum alkaline phosphatase (ALP) (*P* < 0.001) and serum osteocalcin (*P* < 0.01) were significantly higher in the 8E×H8S group as compared to 16S group. After 8 weeks of cessation of exercise and honey supplementation, bone SOS was also significantly higher (*P* < 0.001) in the 8E×H8S group as compared to 16S group. In addition, the serum total calcium (*P* < 0.001), serum ALP (*P* < 0.01), total antioxidant status (TAS) (*P* < 0.01) and glutathione (GSH) (*P* < 0.01) in the 8E×H8S group were significantly higher at the post-test as compared to their respective pre-test values.

**Conclusion:**

These findings demonstrate that there was improved maintenance of the beneficial effects induced by 8 weeks of combined exercise and honey supplementation on bone properties and the antioxidant status after 8 weeks of cessation of exercise and honey supplementation as compared to exercise and honey supplementation alone.

## Introduction

Exercise and nutrition are independently known as important adjustable lifestyle factors necessary for optimal bone health and general wellbeing (1, 2). High peak bone mass is obtained through regular weight-bearing exercises (3). A weight-bearing exercise is defined as a structured, force generating activity performed by the feet on the ground that provides loading to the skeletal region (4). Over the past decade, aerobic dance classes have become one of the most popular activities at fitness clubs and community centers throughout the world. The movements in aerobic dance are associated with ground reaction forces and include a variety of exercises specifically designed to increase muscular strength and power of the lower extremities (5).

Bone metabolism changes can be accessed through biochemical markers namely bone formation markers and bone resorption markers. Bone formation markers include blood total alkaline phosphatase (ALP), bone-specific ALP, serum osteocalcin and procollagen type 1 N propeptide, whereas bone resorption markers include C-terminal telopeptide of type 1 collagen (1CTP), N-terminal telopeptide of type 1 collagen, deoxypyridinoline and pyridinoline (6). There is a close relationship between metabolic bone markers and bone mineral density. Therefore, bone turnover markers can be indirectly used to detect or monitor the early responses of the skeleton to the weight-bearing exercise (7).

During strenuous exercise, the metabolic rate in the skeletal muscle is increased mainly by increased oxygen consumption. This increase in oxygen consumption can lead to an elevation of superoxide onion production and other reactive oxygen species (ROS) in the mitochondria (8). The production of free radicals (FRs), including ROS, has been shown to induce damages in all cellular macromolecules, such as lipids, proteins and DNA (9). On the other hand, the antioxidant system is used to protect an organism from harmful effects of FRs either by forming a less active radical or by quenching the damaging FR chain reaction on substrates (10). The imbalance between FR production and antioxidant defence in favour of the former leads to an oxidative stress state. Oxidative stress is reported to be associated with many diseases and bone degeneration (11). Moreover, oxidative stress and related molecular and cellular changes have been recognised as important factors in the pathogenesis of osteoporosis and age-related bone loss (12). However, the generation of ROS during regular physical exercise might trigger an adaptation in antioxidant capacity to prevent extensive cellular damage (13). Thus, participation in regular physical activity has been shown to enhance the antioxidant status to avoid the deleterious effects of FR production (14).

Besides physical activities, bone health can be enhanced and maintained through adequate nutritional intake. There are potentially numerous nutrients and dietary components that can influence bone health, ranging from macronutrients to micronutrients as well as bioactive food ingredients (15). One of the nutrients that has a long tradition of use within various medical areas and is prescribed for a variety of uses from ancient times is honey. Honey is essentially composed of a complex mixture of carbohydrates and other minor substances, such as organic acids, amino acids, proteins, minerals, vitamins and lipids (16). These chemical constituents of honey make it beneficial for enhancing health (17). It has also been reported that honey has potent antioxidant properties which can help eliminate FRs in the body (18). To date, information on the combined effects of exercise and honey supplementation on antioxidant status is still lacking.

Furthermore, while the effects of increased mechanical loading on the skeletons are obvious, the ability of the skeleton to preserve the exercise induced bone gain after the cessation of exercise is still equivocal. Some studies have suggested that retired athletes who started training in childhood have bone mass benefits that are maintained until adulthood (19, 20). In contrast, other studies reported no positive influence of previous sports participation on bone health (21, 22). Hence, this study investigated the effects of aerobic dance exercise and honey supplementation followed by their subsequent cessation on bone metabolism and antioxidant status in young females.

## Methods

### Participants

Forty-eight healthy female participants with ages ranging from 19 years old–25 years old were recruited among college students. The inclusion criteria included: i) leading a sedentary lifestyle with less than 2 days physical activity per week and ii) did not consume honey regularly prior to the study. The procedures carried out in this study were approved by the Human Research Ethics Committee of Universiti Sains Malaysia.

**Figure 1:**
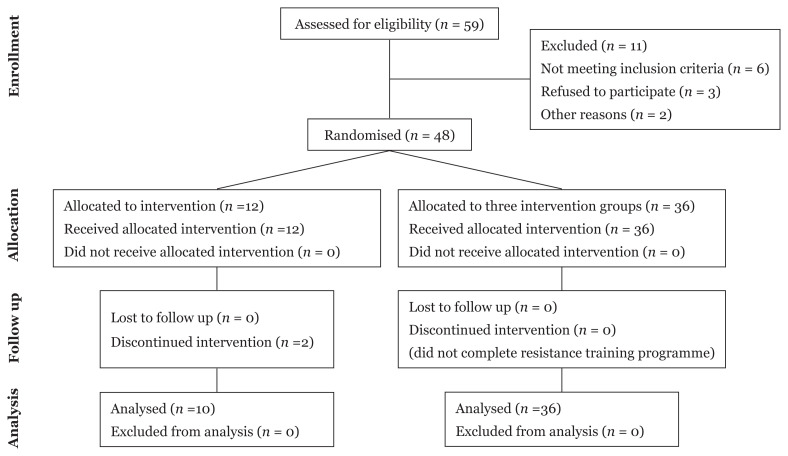
CONSORT diagram showing the flow of participants through each stage of the study

The participants were age- and body weight-matched before they were randomly assigned into four groups with 12 participants in each group, i.e. 16 weeks of sedentary or control group (16S), 8 weeks of aerobic dance exercise followed by 8 weeks of sedentary group (8E×8S), 8 weeks of honey supplementation followed by 8 weeks of sedentary group (8H8S), 8 weeks of combined aerobic dance exercise and honey supplementation followed by 8 weeks of sedentary group (8E×H8S). A CONSORT diagram showing the flow of participants through each stage of this study is illustrated in Figure 1.

### Training Programme

The aerobic dance exercise programme comprised two sessions of high impact and low impact aerobic dance exercise and one session of step board aerobic dance exercise in a week in the 8E×8S and 8E×H8S groups. The 1-h aerobic dance exercise session started with 10 min of warm-up activities, followed by 40 min of either ‘low impact and high impact’ or ‘step board’ movements and ended with 10 min of cooling down. This training programme was conducted three times per week for 8 weeks in the Health Campus, Universiti Sains Malaysia and it was supervised by one of the researchers.

### Honey Supplementation

Tualang honey, which is a local Malaysian honey sponsored by Federal Agriculture Marketing (FAMA) Malaysia, was used in this study. Tualang honey is dark brown in colour and has a pH of 3.55–4.00. The nutritional contents of Tualang honey include fructose (29.6%), glucose (30.0%), sucrose (0.61%), maltose (7.9%), potassium (0.51%), calcium (0.18%) magnesium (0.11%) and sodium (0.26%) (23). This honey was consumed daily for 8 weeks by the participants in the 8H8S and 8E×H8S groups at a dosage of 20 g (24) diluted in 300 mL of plain water (25). In the 8E×H8S group, the participants were required to consume the honey drink 30 min before performing the aerobic dance exercise on exercise days.

### Cessation of Aerobic Dance Exercise and Honey Supplementation

In the 8E×8S group, aerobic dance sessions were terminated after 8 weeks, whereas in the 8H8S group, participants were required to stop consuming honey supplementation after 8 weeks of the study period. In the 8E×H8S group, the aerobic dance sessions were terminated and the participants were also required to stop consuming honey after 8 weeks. In all these three groups, the participants were instructed to be sedentary for another 8 weeks before the post-intervention parameters were measured.

### Blood Sampling

Blood samples were collected at three time points: pre-test (before intervention), mid test (after 8 weeks of intervention) and post-test (after 8 weeks of cessation of intervention). After a 10-h overnight fast, 10 mL of venous blood sample was taken from the antecubital vein of the participants in the morning. The blood was collected into two separate test tubes, i.e. 2 mL into an ethylenediaminetetraacetic acid (EDTA) tube and 8 mL into a plain tube. Blood samples in the EDTA tubes were used for determining the antioxidant status parameters, such as superoxide dismutase (SOD), reduced glutathione (GSH) and reduced glutathione/glutathione oxidised (GSH/GSSG) ratio. Serum from the clotted blood in the plain tubes was obtained by centrifugation (15 min, 4000 rpm, 4 °C; Health-Ratina 46RS, Germany) and used for determining the serum total antioxidant status (TAS), F_2_-isoprostanes and bone metabolism markers such as serum total calcium, serum ALP, serum osteocalcin and serum 1CTP.

### Biochemical Analysis

Serum total calcium was analysed colorimetrically (Hitachi Automatic Analyser 012, Bohringer Mannhaeim, Germany) by using commercially available reagent kits (Randox, UK). Serum ALP was analysed by using an automatic analyser (Hitachi Automatic Analyser 912, Bohringer Mannheim, Germany) with commercially available reagent kits (Randox, ALP UK). Serum osteocalcin was analysed via a commercially available enzyme-linked immunosorbent assay kit (Nordic Bioscience Diagnosis N-MID^TM^ Osteocalcin ELISA, Denmark) and the concentration was determined by using a photometric microplate reader (Molecular Devices; VersaMax tunablemicroplate reader, USA). Serum 1CTP was analysed via a commercially available enzyme immunoassay kit (Creative Diagnostics, 1CTP ELISA, USA) and the concentration was determined by a photometric microplate reader (molecular devices; VersaMax tunable microplate reader, USA).

Serum TAS was measured by using a commercially available antioxidant assay kit (Randox, UK). The activity of SOD was measured via a commercially available antioxidant assay kit (Randox, UK). Serum F_2-_isoprostanes was analysed by using a commercially available enzyme-immunoassay kit (Cayman’s 8-isoprostane EIA, USA) and the concentration was determined by a photometric microplate reader (molecular devices; VersaMax tunable microplate reader, USA). Blood GSH and GSH/GSSG ratio was analysed by using an enzyme-linked immunosorbent assay (ELISA) method and a commercially available GSH/GSSG ratio assay kit (Oxford Biomedical Research, USA) and the concentration was determined by a photometric microplate reader (molecular devices; VersaMax tunable microplate reader, USA).

### Statistical Analysis

The data were analysed using the Statistical Package for the Social Sciences (SPSS) version 20.0. Prior to the main analysis, the data were screened for accuracy, missing value, outliers and basic assumptions. Two-way repeated measures ANOVA was used to test if there were any statistical differences between groups (main effect of group), within groups (main effect of time) and within-between interactions were tested. Between-group analysis regardless of time was performed by interpreting the *P*-value of the *F*-test followed by *post hoc* least significant differences (LSD) multiple comparisons to locate the differences when the repeated measure ANOVA revealed significant differences. A within groups comparison (time effect) was performed after the assumption of sphericity was checked using Mauchly’s test. The assumption was met when Mauchly’s test was not significant (*P* > 0.05). A pairwise comparison with 95% confidence interval (CI) adjustment was done using Bonferroni correction. The within-group percentage differences were presented as well. Differences were considered significant if the *P*-value was less than 0.05. The within between-group (time-treatment interaction) analysis was interpreted by its *P*-value of the *F*-test followed by estimated marginal means.

## Results

### Physical Characteristics of the Participants

Forty-six healthy female collegiate students (mean age of 21.2 ± 1.2 years old) participated in the present study. Two participants from the control group (16S) were unable to continue the programme due to personal reasons during the experimental period. At the pre-test, the mean body height of the participants was 156.7 (4.1) cm, the mean body weight was 56.6 (9.8) kg, the mean body mass index (BMI) was 23.1 (3.8) kg.m^−2^ and the mean body fat percentage was 32.1 (7.3). There were no significant differences between groups in mean age, body height, body weight and body fat percentage prior to the experimental period. Nevertheless, a significant interaction between the experimental groups across the measurements was found for mean body mass (*F* = 2.97, *P* = 0.011), mean BMI (*F* = 2.84, *P* = 0.014) and mean percentage of body fat (*F* = 5.87, *P* < 0.001). After 8 weeks of intervention, the mean body mass, mean BMI and mean percentage of body fat were significantly lower in the 8E×H8S group as compared to 8E×8S group at mid test. After 8 weeks of cessation, the mean BMI and mean percentage of body fat were significantly lower in 8H8S and 8E×H8S groups as compared to 8E×8S group in the post-test. The HRmax of the participants was 195 beats.min^−1^–201 beats. min^−1^ and their mean HR recorded during the exercise sessions ranging from 120 beats.min^−1^− 140 beats.min^−1^.

### Bone Metabolism Markers

**Figure 2:**
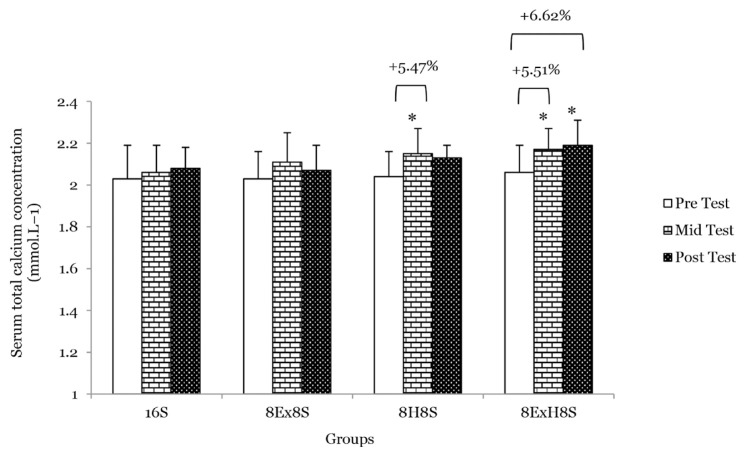
Mean serum total calcium concentration among the groups at pre-, mid- and post-tests Notes:*significantly different from respective pre-test (*P* < 0.05); 16S = 16 weeks of sedentary control group; 8E×8S = 8 weeks of aerobic dance exercise followed by 8 weeks of sedentary group; 8H8S = 8 weeks of honey supplementation followed by 8 weeks of sedentary group; 8E×H8S = 8 weeks of combined aerobic dance exercise with honey supplementation followed by 8 weeks of sedentary group

**Figure 3:**
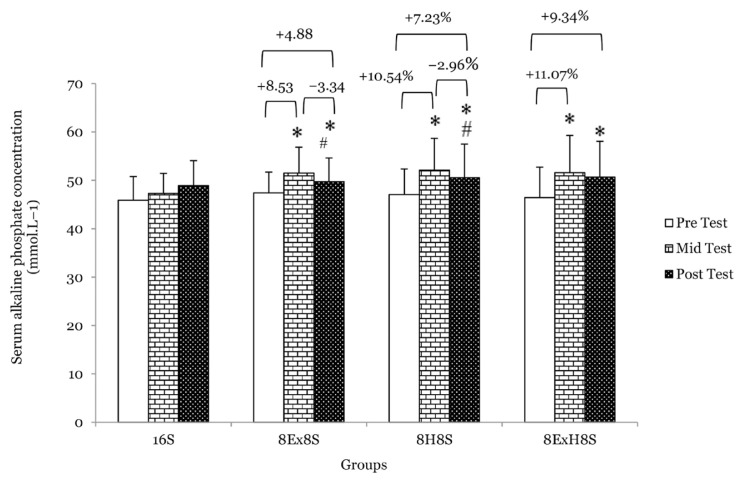
Mean serum ALP concentration among the groups at pre-, mid- and post-tests Notes: *significantly different from respective pre-test (*P* < 0.05); #significantly different from respective mid-test (*P* < 0.05); 16S = 16 weeks of sedentary control group; 8E×8S = 8 weeks of aerobic dance exercise followed by 8 weeks of sedentary group; 8H8S=8 weeks of honey supplementation followed by 8 weeks of sedentary group; 8E×H8S = 8 weeks of combined aerobic dance exercise with honey supplementation followed by 8 weeks of sedentary group

There were no main effects of group on serum total calcium, serum ALP and serum 1CTP. Similarly, there were no significant interaction between the experimental groups across the measurements for serum total calcium and serum osteocalcin. There was a main effect of time on serum ALP (*F* = 47.18, *P* < 0.001). At the mid test, after 8 weeks of intervention, the mean serum ALP concentrations were significantly higher in 8E×8S, 8H8S and 8E×H8S groups as compared to 16S group at mid test (Table 1). There was also a main effect of time on serum total calcium (*F* = 17.42, *P* < 0.001). The mean serum total calcium concentration increased significantly by 5.5% in the 8H8S group (*P* = 0.024) and 5.5% in the 8E×H8S group (*P* = 0.016) at the mid test and by 6.6% in the 8E×H8S group (*P* = 0.001) at the post-test when compared to their respective pre-test values (Figure 2). The percentage increment in serum ALP, a bone formation marker in the 8E×H8S group was the highest at the mid test (11.1%) (*P* < 0.001) and at the post-test (9.3%) (*P* = 0.015) compared to the other experimental groups (Figure 3).

**Figure 4:**
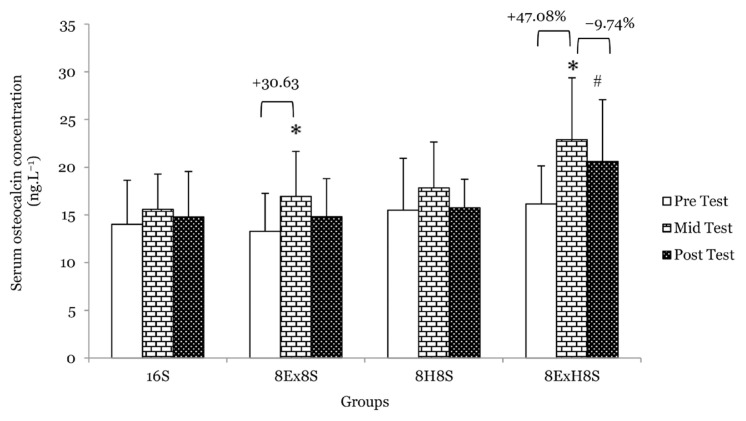
Mean serum osteocalcin concentration among the groups at pre-, mid- and post-tests Notes: *significantly different from respective pre-test (*P* < 0.05); #significantly different from respective mid-test (*P* < 0.05); 16S = 16 weeks of sedentary control group; 8E×8S = 8 weeks of aerobic dance exercise followed by 8 weeks of sedentary group; 8H8S = 8 weeks of honey supplementation followed by 8 weeks of sedentary group; 8E×H8S = 8 weeks of combined aerobic dance exercise with honey supplementation followed by 8 weeks of sedentary group

**Figure 5:**
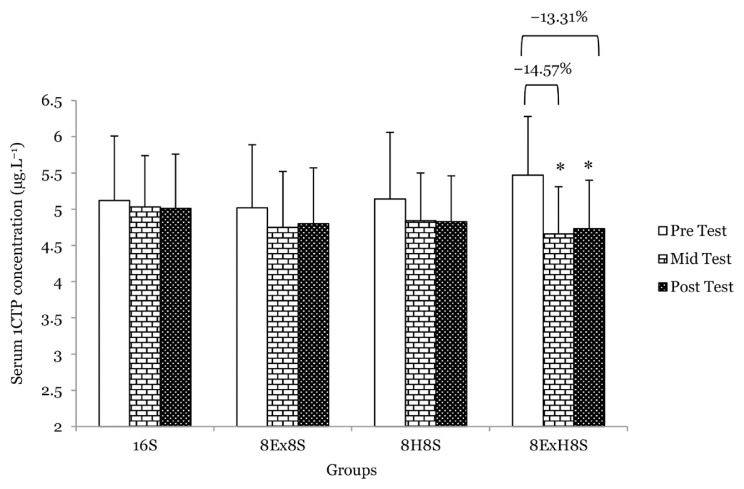
Mean serum 1CTP concentration among the groups at pre-, mid- and post-tests Notes: *significantly different from respective pre-test (*P* < 0.05); 16S = 16 weeks of sedentary control group; 8E×8S = 8 weeks of aerobic dance exercise followed by 8 weeks of sedentary group; 8H8S = 8 weeks of honey supplementation followed by 8 weeks of sedentary group; 8E×H8S = 8 weeks of combined aerobic dance exercise with honey supplementation followed by 8 weeks of sedentary group

There was a main effect of time on serum osteocalcin (*F* = 17.70, *P* < 0.001). The serum osteocalcin increased by 30.6% in the 8E×8S group (*P* = 0.002) and 47.1% in the 8E×H8S group (*P* = 0.018) at the mid test, then it dropped 9.7% at the post-test (*P* = 0.012) as compared to pre-test (Figure 4). There was also a main effect of time on serum 1CTP (*F* = 12.28, *P* < 0.001). A significant reduction in serum 1CTP, a bone resorption marker, was observed in the 8E×H8S group, namely by 14.6% at the mid test (*P* < 0.001) and −13.3% at the post-test (*P* < 0.001) when compared to during pre-test (Figure 5).

### Antioxidant Status

**Figure 6:**
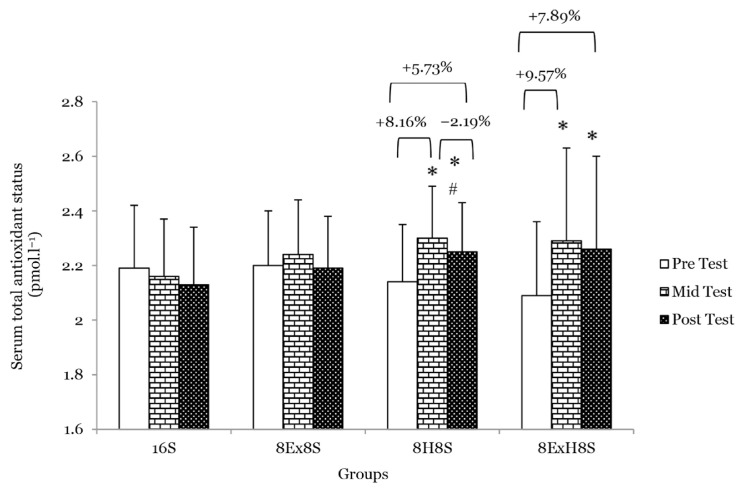
Mean serum total antioxidant status among the groups at pre-, mid- and post-tests Notes: *significantly different from respective pre-test (*P* < 0.05); #significantly different from respective mid-test (*P* < 0.05); 16S = 16 weeks of sedentary control group; 8E×8S = 8 weeks of aerobic dance exercise followed by 8 weeks of sedentary group; 8H8S = 8 weeks of honey supplementation followed by 8 weeks of sedentary group; 8E×H8S = 8 weeks of combined aerobic dance exercise with honey supplementation followed by 8 weeks of sedentary group

**Figure 7:**
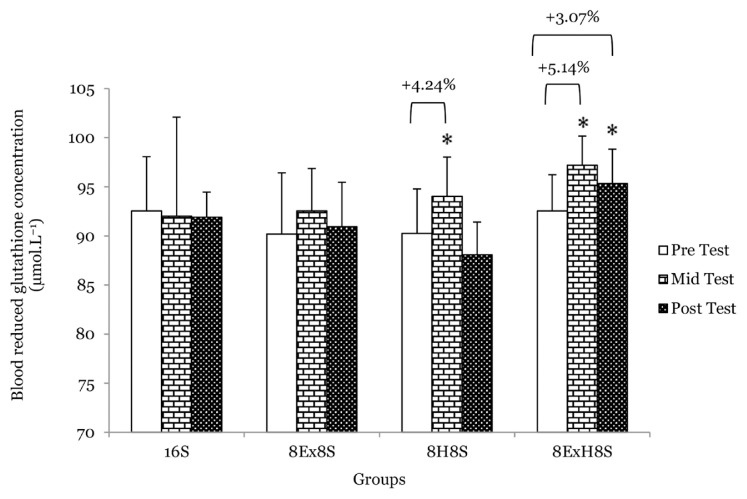
Mean blood reduced GSH concentration among the groups at pre-, mid- and post-tests Notes: *significantly difference from respective pre-test (*P* < 0.05); 16S = 16 weeks of sedentary control group; 8E×8S = 8 weeks of aerobic dance exercise followed by 8 weeks of sedentary group; 8H8S = 8 weeks of honey supplementation followed by 8 weeks of sedentary group; 8E×H8S = 8 weeks of combined aerobic dance exercise with honey supplementation followed by 8 weeks of sedentary group

**Figure 8:**
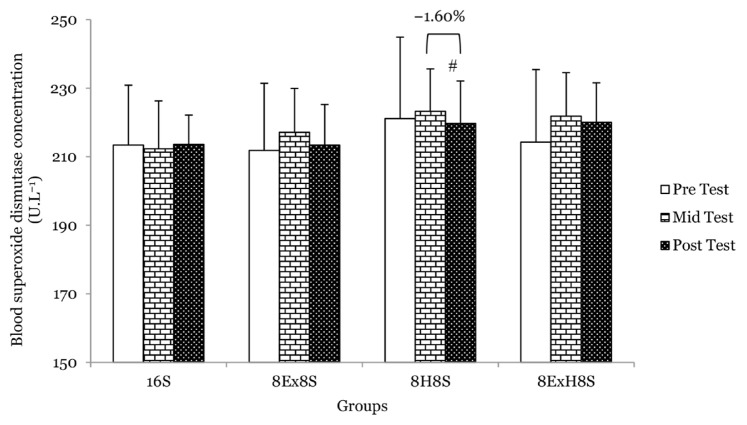
Mean blood superoxide dismutase concentration among the groups at pre-, mid- and post-tests Notes: #significantly different from respective mid-test (*P* < 0.05); 16S = 16 weeks of sedentary control group; 8E×8S = 8 weeks of aerobic dance exercise followed by 8 weeks of sedentary group; 8H8S = 8 weeks of honey supplementation followed by 8 weeks of sedentary group; 8E×H8S = 8 weeks of combined aerobic dance exercise with honey supplementation followed by 8 weeks of sedentary group

There were no main effects of group on serum TAS, blood SOD and GSH between groups at the mid-test and post-test. No significant interaction between the experimental groups across the measurements was found for parameters related to the antioxidant status. There was a main effect of time on serum TAS (*F* = 11.15, *P* < 0.001). The within-group comparison revealed that the serum TAS significantly increased in the 8H8S group by 8.2% at the mid-test (*P* = 0.040) and 5.7% at the post-test (*P* = 0.019) as compared to the pre-test. Similarly, in the 8E×H8S group, the serum TAS was found to be 9.6% higher at the mid test (*P* = 0.001) and 7.9% higher at the post-test (*P* = 0.009) as compared to the pre-test (Figure 6). There was a main effect of time on blood GSH (*F* = 7.91, *P* < 0.001). The mean blood GSH concentration compared to the pre-test increased significantly by 4.2% (*P* < 0.001) in the 8H8S group and by 5.1% at the mid test (*P* = 0.001) and 3.1% at the post-test (*P* = 0.018) in the 8E×H8S as compared to pre-test (Figure 7). There was no significant change in blood SOD at the mid- and post-test when compared to the pre-test (Figure 8).

### Oxidative Stress Markers

**Figure 9:**
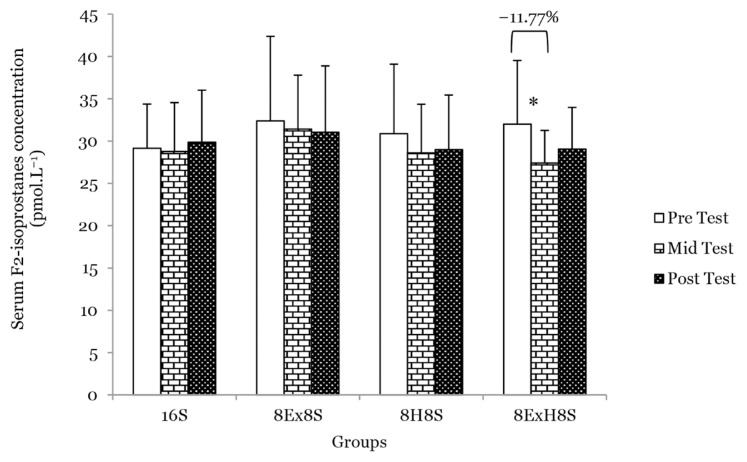
Mean serum F2-isoprostanes concentration among the groups at pre-, mid- and post-tests Notes: *significantly different from respective pre-test (*P* < 0.05); 16S = 16 weeks of sedentary control group; 8E×8S = 8 weeks of aerobic dance exercise followed by 8 weeks of sedentary group; 8H8S = 8 weeks of honey supplementation followed by 8 weeks of sedentary group; 8E×H8S = 8 weeks of combined aerobic dance exercise with honey supplementation followed by 8 weeks of sedentary group

There was a main effect of time on F_2_- isoprostanes (*F* = 5.53, *P* = 0.007). The mean serum F_2_-isoprostanes concentration decreased significantly in the 8E×H8S group (*P* = 0.030) by 11.8% at the mid test as compared to the pre-test (Figure 9).

## Discussion

Changes in bone mass usually occur at a slow rate, and measurement of bone mineral density (BMD) may not be adequate to detect acute changes in bone metabolism as a result of physical activity. Thus, the skeletal response to exercise can be measured by using blood biochemical markers to estimate the bone remodeling rate by comparing the resorption markers to formation markers. According to Alghadir et al. (26), bone biochemical markers can also reflect the cellular activities of bone formation and resorption, and they are useful tools to investigate the mechanisms of exercise-induced changes in bone mass.

In the present study, there were increases in serum ALP and serum osteocalcin (bone formation markers), and also a decrease in serum 1CTP (bone resorption marker), in the 8E×8S group at mid-test as compared to their respective baseline measurements. It was reported by Duncan and Turner (27) that mechanical loading via physical activity can inhibit bone resorption and increase bone formation. Increases in bone formation markers, such as serum osteocalcin and serum ALP due to exercise as observed in the present study were also reported by Menkes et al. (28) where serum osteocalcin and serum ALP increased after 16 weeks of strength training in older men. An observation of an increase in bone formation marker, namely serum ALP and serum osteocalcin in young males, had also been reported following five weeks of endurance exercise in adolescent males (29).

Alghadir et al. (26) reported that serum bone specific ALP, serum osteocalcin and free calcium in healthy elderly participants can be increased by performing moderate intensity circuit training using treadmill, bicycle and staircase (3 sessions per week for 12 weeks). However, unlike the results reported by Alghadir et al. (26), no significant changes in serum total calcium were observed after 8 weeks of aerobic dance in the present study. This absence of changes in serum total calcium after 8 weeks of aerobic dance exercise was similar to the findings of Maimoun et al. (30) where no significant changes in serum calcium were observed in response to cycling.

The reduction in serum 1CTP after 8 weeks of aerobic dance exercise in the 8E×8S group as observed in the present study was not observed by Rahim et al. (31), who reported that aerobic dance exercise significantly elevated the concentration of such bone resorption marker. The age of the participants might be the reason for the contrasting findings between the present study and Rahim et al. (31). Young females were recruited in the present study, whereas Rahim et al. (31) study was conducted on adult women. These observations highlight that the bone response to exercise training may be age dependent. It is speculated that increase in serum ALP and serum osteocalcin and decrease in serum 1CTP after 8 weeks of aerobic dance exercise observed in the present study may reflect the enhancement in bone re-modelling in favour of greater BMD achievement compared to the sedentary control.

Regarding the effects of nutritional supplementation on bone, honey was used as a supplementation in the present study based on the hypothesis that natural honey contains a wide range of nutrients such as carbohydrates, protein, vitamin D, vitamin K, calcium, phosphorous, magnesium and flavonoids (32). Honey has been recommended to promote bone health by increasing the bioavailability of calcium and other nutrients (17). Honey acts as a carrier to facilitate the transportation and absorption of those nutrients, particularly calcium, in the digestive system. In addition, it has been reported that Malaysian Tualang honey has the potential to be used as an alternative treatment for postmenopausal osteoporosis due to its antioxidative and anti-inflammatory properties against bone loss (17).

The serum total calcium and serum ALP increased significantly after 8 weeks of 20 g/day of Tualang honey supplementation as compared to the baseline measurements. It is believed that the effect of nutritional supplementation on bone is through the remodeling process involving the interaction between the markers of bone formation and resorption with the endocrine hormones. A higher calcium load could lead to a decline in parathyroid hormone, thereby reducing the level of bone resorption markers in the blood. Besides the improvement in serum total calcium and serum ALP following 8 weeks of honey supplementation, there were no significant changes in serum osteocalcin, another type of more specific bone formation marker and serum 1CTP, as a bone resorption marker. It is speculated that the dosage of honey prescribed in the present study may not be adequate to increase serum osteocalcin and reduce serum 1CTP concentrations during the 8 weeks study period.

The present study also provided evidence of greater beneficial effects of combined exercise and nutritional supplementation in enhancing bone health. The serum osteocalcin was significantly higher when aerobic dance exercise was combined with honey supplementation for 8 weeks in the 8E×H8S group as compared to the control group. The serum ALP was significantly higher in the 8E×H8S group as compared to the control group. The interaction between physical activity and nutrition was explained in a study on infants by Specker et al. (33), who reported that bone response to activity was dependent on the calcium intake of the infants. The findings of the present study were supported by Ooi et al. (24) who reported that 6 weeks of aerobic dance exercise combined with honey supplementation exhibited the highest percentage of the increment in serum ALP among young Malaysian females.

The serum total calcium, serum ALP and serum osteocalcin increased, and the serum 1CTP decreased at the mid-test as compared to pre-test in the 8E×H8S group. These results imply that a combination of aerobic dance and honey supplementation may enhance bone metabolism in young females. Daly and Petit (34) stated that exercise may interact with nutrients, for example calcium, vitamin D and protein, to enhance bone formation. However, Evans (35) reported that there were no apparent additive effects of soy combined with exercise on the markers of bone turnover. The differences in the gender of the participants, types of exercise and nutritional supplementation prescribed, could explain the discrepancy between the findings of these studies. The reasons for greater beneficial bone effects in the 8E×H8S group could be attributed to the enhancement in blood flow to the exercising muscle by dynamic loading during the aerobic dance exercise sessions, as well as the increased availability of nutrients such as calcium and flavonoids, in the honey consumed.

While the beneficial effects of exercise on bone are quite well established. However, it is not clear whether these benefits will be maintained after cessation of training or a decreased level of physical activity. In the present study, our data indicate that there were no significant differences in bone metabolism markers between the groups after 8 weeks of cessation of the exercise programme. It was found that serum ALP was significantly higher as compared to its baseline value even after the cessation of exercise. The higher level of serum ALP at the post-test in the 8E×8S group implies that the bone formation process may still be active after 8 weeks of cessation of the aerobic dance exercise. Bass et al. (36) reported that the starting age of training is the key factor in the maintenance of previous bone gain and part of the exercise-induced bone gain that is obtained during the years of growth may persist despite the decreased physical activity. The reason for the lack of full maintenance of all the measured bone parameters after 8 weeks of cessation of aerobic dance exercise in the present study could be attributed to the age of the participants. The participants in the present study were young female adults with ages ranging from 19 years old to 25 years old. Hence, they may have passed the fast bone growth and development period. The intensity and duration of the aerobic dance exercise prescribed in the present study can also probably contribute to the lack of maintenance in the measured bone parameters. Therefore, an exercise programme with higher intensity and longer duration may be warranted in order to achieve better maintenance of bone parameters.

After 8 weeks of cessation of honey supplementation, there was no significant difference in serum total calcium between the mid-test and the post-test in the 8H8S group. This finding implies that the beneficial effects of 8 weeks of honey supplementation can still be maintained after 8 weeks of cessation of honey supplementation. On the other hand, after 8 weeks of cessation of honey supplementation at the post-test, the serum ALP concentration was significantly lower than the mid-test, nevertheless, it was still significantly higher than the baseline value. This data indicates that the improvement in serum ALP following 8 weeks of honey supplementation was sustained even after 8 weeks of honey supplementation cessation. For serum osteocalcin and serum 1CTP, there were no significant differences between the pre-, mid- and post-tests in the 8H8S group. These results demonstrate that 8 weeks of honey supplementation followed by 8 weeks of honey supplementation cessation did not result in any significant changes in serum osteocalcin, a bone formation marker and serum 1CTP, a bone resorption marker in young females. In another study on calcium and vitamin D supplementation, Dawson-Hughes et al. (37) reported that the bone turnover rates, as measured by the serum osteocalcin level, returned to their original concentrations within the 2-year follow-up period in both men and women. The mechanism responsible for reversing the bone remodeling process after withdrawal of calcium load is not fully understood. However, it can be speculated that after the supplementation was withdrawn, the bone remodeling rate increased and consequently additional bone mineral deposited during the supplementation phase was mobilised when the supplementation was discontinued, and the benefits on bone status therefore disappeared (38).

Adequate maintenance of bone turnover markers was also observed after 8 weeks of cessation of aerobic dance exercise and honey supplementation in the 8E×H8S group. This is based on the fact that the serum total calcium and serum ALP remained significantly higher at the post-test as compared to its pre-test values. After 8 weeks of cessation period in the 8E×H8S group, the serum 1CTP, a bone resorption marker, was still lower in the post-test than in the pre-test. These results indicate that the beneficial effects of 8 weeks of combined aerobic dance exercise and honey supplementation on increasing serum total calcium and serum ALP and reducing serum 1CTP can be maintained after 8 weeks of cessation of aerobic dance exercise and honey supplementation. However, the serum osteocalcin reduced significantly at the post-test in comparison with the mid-test, which implying poor maintenance of serum osteocalcin after 8 weeks of cessation in the 8E×H8S group. Although the potential mechanism behind the exercise-nutrition interaction is not fully understood, the findings of the present study are in favour of the synergistic effect of combined exercise and nutritional supplementation to better maintain the beneficial bone effect from aerobic dance exercise and honey supplementation.

In the present study, the markers of antioxidant status and oxidative stress were not significantly different after 8 weeks of intervention among the four experimental groups. It may indicate that 8 weeks of aerobic dance exercise conducted in the present study did not disturb the balance between the oxidant and antioxidant systems. It is reported that acute exercise of sufficient volume, intensity and duration can increase reactive oxygen and nitrogen species (RONS) production, which can lead to the oxidation of several biological molecules. However, repeated exposure to increased RONS production from chronic exercise training leads to an upregulation in the endogenous antioxidant system. This provides adaptive protection from RONS during subsequent training sessions, as well as during non-exercise related conditions (39).

It was reported by Robertson et al. (40) that the increase in the blood antioxidant defence system in response to exercise is associated with endurance training among trained runners. On the other hand, exhaustive exercise appears to disrupt the antioxidant system as reported by Tartibian and Maleki (41) that 8 weeks of intense cycling training significantly increased markers of oxidative stress and resulted in a reduction in antioxidant capacity of healthy professional male road cyclists. However, enhancements in antioxidant activity with reduction in oxidative stress markers were observed by Tartibian and Maleki (41) when honey supplementation was combined with cycling exercise. In the present study, no significant differences in TAS were observed between the experimental groups at the mid-test and post-test.

In the present study, after 8 weeks of honey supplementation, the TAS, SOD and GSH were significantly higher than their baseline measurement, indicating the enhancement of the endogenous antioxidant system. In the first report on the antioxidant properties of Malaysian honeys, Aljadi and Kamaruddin (42) reported that Gelam honey and Coconut honey have antioxidative and radical scavenging properties due to their phenolic content. Similarly, the antioxidant properties of Malaysian Tualang honey have also been documented by Mohamed et al. (43). These authors confirmed that the antioxidant properties of Malaysian Tualang honey are similar to other types of honeys reported in the literature. Because phenolic content and flavonoids have been reported to have antioxidant properties (44, 45), this can be the reason for the improvement in the TAS after 8 weeks of honey supplementation in the present study. Supporting the findings of the present study, Ibrahim Khalil et al. (46) also reported that among Malaysian honey samples, Tualang honey is the richest in phenolic acids and flavonoid compounds, which have strong FR-scavenging activities.

Physical activity is associated with an increase in body oxygen uptake and during exhaustive exercise, the generation of ROS elevates to the level that overcomes the antioxidant defence system (47). One of the adaptations of regular physical activity is the upregulation of the endogenous antioxidant status. However, one of the findings of the present study is that oxidative stress was not observed after 8 weeks of aerobic dance exercise either with or without honey supplementation where the level of F_2_-isoprostanes did not change significantly after 8 weeks of intervention. Finaud et al. (48) reported that FR generation may not occur with low exercise intensity. In such situations, the antioxidant capacity is not over-reached and FR-induced damage does not appear. Therefore, it can be explained that the exercise regimen prescribed in the present study did not disturb the balance between the oxidant and antioxidant systems. In addition, this finding can also be attributed to the enhanced antioxidant system following exercise training and/or from the intake of honey which has antioxidant properties.

Another notable finding of the present study indicates that there was a greater improvement in antioxidant status following the combination of aerobic dance and honey supplementation. The serum TAS and blood GSH increased and F_2_-isoprostane decreased after 8 weeks of intervention in the 8E×H8S group. Similarly, no sign of oxidative stress marker was observed by Mastaloudis et al. (49) when vitamin C and E were supplemented to runners and no significant changes in F_2_-isoprostanes were observed.

Aerobic dance exercise and honey supplementation for 8 weeks, however, did not seem to have any effect on SOD. Similar findings have been reported where untrained individuals did not show an increase in muscle SOD activity after completing 8 weeks moderate-intensity cycling exercise programme (50). On the other hand, higher SOD activity was observed in sprinters, runners and volleyball athletes (51, 52) than untrained individuals, suggesting that an increased level of SOD activity in response to exercise intervention is more common in trained individuals.

## Conclusion

The findings of the present study indicate that there was an improved maintenance of serum total calcium, serum ALP, serum TAS and blood reduced GSH when aerobic dance exercise was combined with honey supplementation. Hence, the combination of aerobic dance exercise with honey supplementation might be effective for the preservation of exercise-induced enhancement in bone health and antioxidant status.

## Figures and Tables

**Table 1:** Descriptive statistics for serum ALP concentration

Serum ALP concentration (mmol.L^−1^)								

Variables	16S		8E×8S		8H8S		8E×H8S	
			
	Mean (SD)	95% CI	Mean (SD)	95% CI	Mean (SD)	95% CI	Mean (SD)	95% CI
Pre-test	45.90 (4.89)	42.55, 49.25	47.42 (4.29)	44.36, 50.48	47.08 (5.26)	44.02, 50.14	46.42 (6.30)	43.36, 49.48
Mid-test	47.30 (4.14)	43.36, 51.24	51.50& (5.35)	47.90, 55.10	52.08& (6.60)	48.49, 55.68	51.58& (7.70)	47.99, 55.18
Post-test	48.90 (5.17)	44.92, 52.88	49.75 (4.88)	46.12, 53.38	50.58 (6.91)	46.95, 54.21	50.67 (7.40)	47.04, 54.30

Notes:

& significantly different (*P* < 0.05) from 16S at mid-test; 16S = 16 weeks of sedentary control group; 8E×8S = 8 weeks of aerobic dance exercise followed by 8 weeks of sedentary group; 8H8S = 8 weeks of honey supplementation followed by 8 weeks of sedentary group; 8E×H8S = 8 weeks of combined aerobic dance exercise with honey supplementation followed by 8 weeks of sedentary group
